# Engineering plant holobionts for climate-resilient agriculture

**DOI:** 10.1093/ismejo/wraf158

**Published:** 2025-08-01

**Authors:** Nayanci Portal-Gonzalez, Wenbo Wang, Wenxing He, Ramon Santos-Bermudez

**Affiliations:** School of Biological Science and Technology, University of Jinan, No. 336, West Road of Nan Xinzhuang, Jinan, Shandong 250022, China; School of Biological Science and Technology, University of Jinan, No. 336, West Road of Nan Xinzhuang, Jinan, Shandong 250022, China; School of Biological Science and Technology, University of Jinan, No. 336, West Road of Nan Xinzhuang, Jinan, Shandong 250022, China; School of Biological Science and Technology, University of Jinan, No. 336, West Road of Nan Xinzhuang, Jinan, Shandong 250022, China

**Keywords:** plant holobiont, synthetic microbial communities, microbiome engineering, microbial ecology, keystone taxa, synthetic biology, root microbiome, systems agriculture

## Abstract

The plant holobiont—an integrated unit of the host and its microbiome—has co-evolved through ecological and genetic interactions. Microbiome engineering offers a promising route to enhance resilience in response to climate stress, soil degradation, and yield stagnation. This review presents an integrated framework combining microbial ecology, synthetic biology, and computational modeling to rationally design synthetic microbial communities (SynComs) for agriculture. We outline ecological principles—priority effects, keystone taxa, and functional redundancy—that shape microbiome assembly and guide SynCom design. Strategies like CRISPR interference, biosensor circuits, and quorum-sensing modules enable programmable microbial functions. We also highlight the predictive potential of in silico modeling—including genome-scale metabolic models, dynamic flux balance analysis, and machine learning—to simulate interactions, optimize SynCom composition, and enhance design accuracy. To bridge lab and field, we discuss native microbial chassis, encapsulation, and precision delivery as tools for scalable, ecosystem-integrated deployment. We introduce the concept of the *programmable holobiont*: an engineered plant-microbe partnership capable of dynamic feedback, interkingdom signaling, and ecological memory. This systems-level perspective reframes plants as designable ecosystems. By synthesizing cross-disciplinary advances, we offer a roadmap for climate-resilient agriculture, where engineered microbiomes improve sustainability, yield stability, and environmental adaptation.

## Introduction

The *plant holobiont* concept, introduced by Vandenkoornhuyse et al. [1], reshapes our understanding of plants as ecological collectives influenced by their co-evolving microbial partners. This perspective has transformed the field of plant biology and opened up exciting opportunities for intervention [2]. As agricultural systems confront compounding stresses—including global warming [3], declining rainfall, increased drought frequency, shrinking arable land, and a growing global population—there is an urgent need to harness the microbiome as a lever for sustainability and resilience [4, 5]. *Microbiome engineering* offers promising solutions, such as enhancing nutrient uptake [6], conferring pathogen resistance [7], and reducing reliance on agrochemicals [8].

We synthesize advances in microbial ecology, synthetic biology, and computational modeling to inform the rational design of synthetic microbial communities (SynComs). Emphasis is placed on ecological rules, programmable circuits, and in silico simulations for building climate-resilient plant–microbe systems. We further introduce a unifying framework: the *programmable holobiont*—an engineered plant–microbe alliance capable of adaptive behavior, interkingdom signaling, and ecological memory. This reframing sees plants as co-designers of their microbiota, with synthetic ecology offering tools to modulate interactions. Innovations such as *microbial memory* and *chassis design* position microbiome engineering as a transformative frontier in biology.

This review *aims* to synthesize current knowledge on how plants interact with beneficial and pathogenic microbes and how these insights can be translated into microbiome engineering strategies. We compare microbial strategies across the mutualism–pathogenicity spectrum and how these interactions can inform the *rational design of SynComs* and *programmable holobionts*. By integrating advances in microbial ecology, synthetic biology, and computational modeling, we offer a roadmap toward designing plant holobionts that are resilient, adaptive, and scalable for future agricultural challenges.

The review is structured as follows: *Section 2* introduces microbiome assembly rules. *Section 3* discusses SynCom design and field translation. *Section 4* covers computational modeling. *Section 5* explores synthetic biology tools. *Section 6* introduces programmable holobionts. *Sections 7–8* cover behavioral control and chassis microbes. *Sections 9–12* present conceptual frontiers, including interkingdom communication and co-evolution. *Sections 13–15* discuss constraints, future directions, and a translational roadmap. *Section 16* concludes with a vision of plants as designable ecosystems. Supplementary Table S1 defines key terms used throughout the document.

## Assembly rules: from ecology to engineering

Microbiome assembly in the rhizosphere follows foundational ecological processes: *selection*, *dispersal*, *drift*, and *diversification* [9–11]. These forces operate across spatial and temporal scales, shaping plant-associated microbiota's taxonomic and functional profiles [12]. Initial assembly occurs in the bulk soil, where edaphic factors, land use, and biogeographic history shape the local species pool. Host genotype, developmental stage, and exudate composition at the root interface are selective filters [11].

*Priority effects*—the influence of microbial arrival order on community structure—can yield divergent states even under identical conditions [13, 14]. Even under similar environmental conditions, *stochastic variati*on in colonization can produce divergent community states [12]. In the context of SynComs, such priority effects may hinder reproducibility or be harnessed to guide deterministic assembly. Thus, classical *ecological dynamics* become engineering levers.

Inter-microbial interactions—ranging from antagonism to cooperation—strongly affect community assembly. Competitive exclusion, syntrophic partnerships, and cross-feeding mediate chemical signaling, resource competition, and niche complementarity [15]. Unraveling these relationships is crucial for designing stable, multifunctional SynComs. Advances in *gnotobiotic systems*, spatial *metabolomics*, and single-cell *omics* now enable precise mapping of such interactions.

Plants recruit microbes via non-random chemical signaling, primarily through exudates rich in sugars, amino acids, and secondary metabolites [16]. Genetic loci linked to root architecture, immunity, and metabolism correlate with microbiome profiles, indicating a heritable core [17]. For example, *Medicago truncatula* secretes luteolin to attract nitrogen-fixing *Sinorhizobium meliloti* [18]. Such findings suggest that hosts actively shape their microbial assemblages.

Microbiomes comprise a *core fraction* (10%–20%) tied to host genotypes and essential functions (e.g. nitrogen fixation) and an *accessory fraction* (80%–90%) more variable across environments [19, 20]. Although *keystone taxa* are frequently inferred from co-occurrence networks, these correlations may overstate their ecological relevance. Functional redundancy and context dependence challenge the idea of universal keystones [21]. Function-based, rather than taxon-based, design—focusing on metabolic traits and ecological roles—offers a more stable engineering foundation.

**Figure 1:**
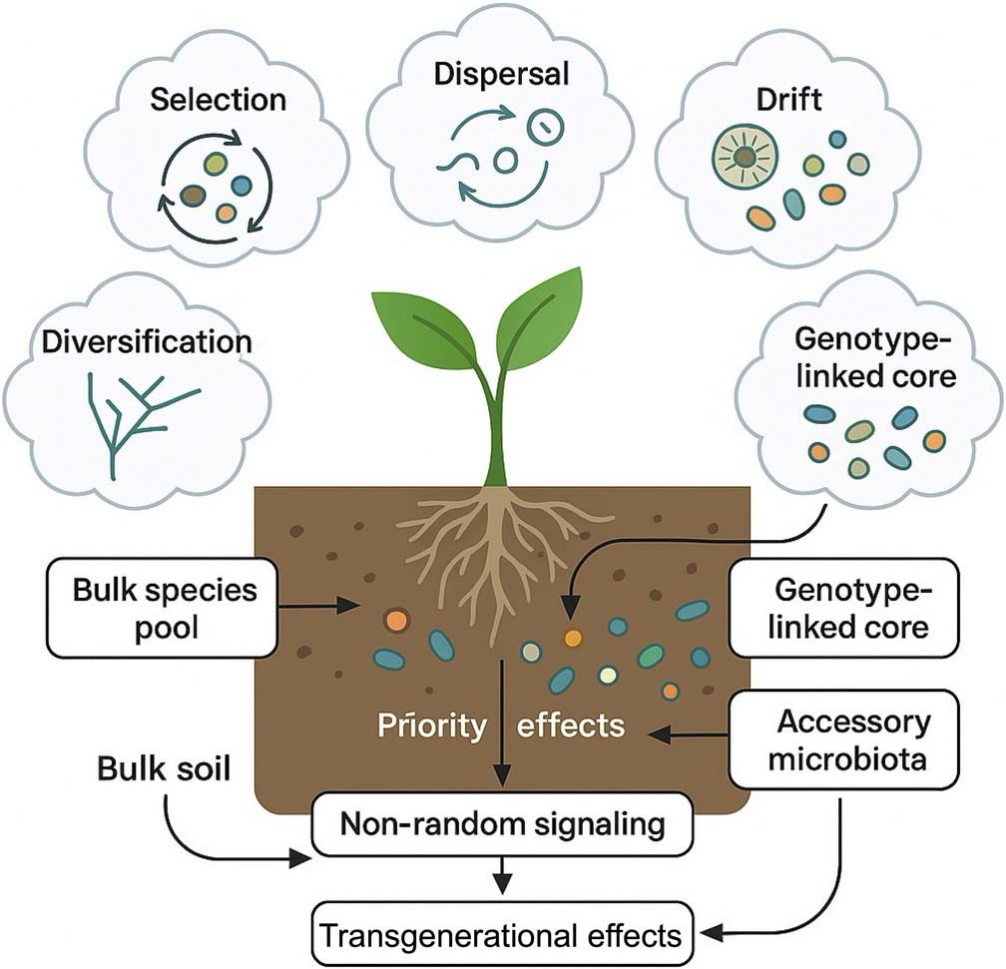
Ecological principles guiding rhizosphere microbiome assembly and engineering. Microbiome assembly in the rhizosphere is shaped by ecological forces such as selection, dispersal, drift, and diversification across spatial and temporal scales. Initial assembly begins in the bulk soil, with edaphic factors, land use, and biogeographic history determining the species pool. The host genotype, developmental stage, and exudate composition at the root-soil interface act as selective filters. Microbial priority effects and inter-microbial interactions (e.g. competition, cooperation, cross-feeding) influence community structure and stability. Plants non-randomly recruit microbes via specialized root exudates, establishing a heritable core microbiome and a context-dependent accessory community. Vertical transmission and legacy effects from seed and soil contribute to transgenerational assembly patterns. These dynamics provide an ecological foundation for microbiome engineering and breeding strategies that leverage host-microbe co-selection and functional trait-based design.

Assembly spans generations. Vertical transmission of endophytes, seed microbiomes, and transgenerational soil feedbacks introduces *legacy effects* that influence holobiont structure over time [22]. These dynamics create opportunities for microbiome-assisted breeding by integrating microbial heritability into crop improvement, grounded in foundational ecological principles of inheritance, selection, and microbial coadaptation (Fig. 1).

## Designing SynComs: from concepts to field

*Synthetic communities* (SynComs) are engineered microbial consortia designed to test or promote specific host traits. As microbiome research shifts from descriptive to functional approaches, SynComs offers a controlled framework for understanding and directing plant-microbe interactions. Yet, rational design must balance simplicity for reproducibility with sufficient complexity to retain ecological function.

Key performance factors include *functional redundancy*, *niche complementarity*, and *emergent properties* [23, 24]. Oversimplified SynComs may lack essential interactions, while overly complex ones hinder mechanistic understanding. Trade-offs should align with the intended application—mechanistic insight versus field deployment. *Trait-based designs* can optimize outcomes by assembling microbes such as nitrogen fixers, hormone producers, or pathogen antagonists.

**Figure 2:**
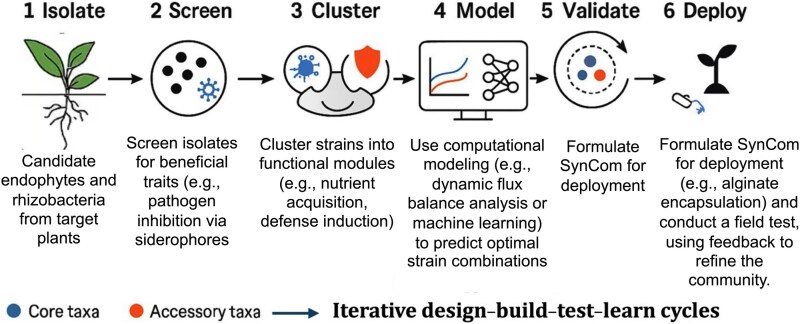
Workflow for rational SynCom design and deployment. The six-step pipeline outlines the iterative engineering of synthetic microbial communities (SynComs): (1) Isolate candidate endophytes and rhizobacteria from target plants or resilient environments. (2) Screen isolates for beneficial traits such as siderophore production, hormone modulation, nitrogen fixation, or pathogen inhibition. (3) Cluster strains into functional modules (e.g. nutrient acquisition, defense induction), and classify as core or accessory taxa. (4) Model optimal combinations using computational tools, including dynamic flux balance analysis, genome-scale metabolic models, or machine learning. (5) Validate SynCom performance in controlled systems (e.g. EcoFABs), tracking colonization, host response, and inter-strain interactions. (6) Deploy SynComs using delivery systems such as alginate encapsulation or seed coating, followed by field trials. Feedback at each stage supports iterative design–build–test–learn (DBTL) refinement cycles for ecological robustness. Each step of the pipeline is addressed in a dedicated section of the review: Steps 1–3 in Section 3, Step 4 in Section 4, and Steps 5–6 in Sections 5–6 and 13.

The *origin of strains* is also critical. Indigenous microbes from native soils typically outcompete lab strains due to better ecological adaptation [25, 26]. Formulations should prioritize ecological fitness, colonization ability, and metabolic compatibility [27]. Recent efforts integrating metagenomic profiling and trait curation have yielded minimal yet robust SynComs with field-like resilience [28], following a stepwise design pipeline from *strain identification to deployment* (Fig. 2).

Despite promising prototypes, many SynComs fail in complex soils [29]. This underscores the importance of *priority effects*, *guild structure*, and interactions with native microbiota. Strategies like *soil pre-conditioning*, *host exudates modulation*, and *helper strain co-inoculation* are emerging to enhance persistence and performance.

SynCom development must follow iterative *design–test–refine cycles*. Integrated pipelines combining *omics*, ecological modeling, and trait-based selection make SynComs increasingly predictive and field-ready, expanding their use beyond model systems into real-world agriculture.

This section discusses steps 1–3 of the SynCom design pipeline (as shown in Fig. 2), focusing on strain isolation, trait screening, and selection logic. Step 4—computational modeling—is explored in detail in Section 4 due to its emerging role in predictive design. Steps 5–6, including validation, formulation, and deployment, are addressed in Sections 5 through 6 and later revisited in the context of field translation in Section 13.

## Computational Modeling and In Silico SynCom design

Among the steps in the SynCom design pipeline, *computational modeling* (*Step 4 in*  Fig. 2) is a critical conceptual and technical frontier. While earlier steps—such as strain selection and functional screening—are increasingly standardized, modeling approaches remain relatively underutilized despite their transformative potential. As microbial consortia design grows more complex, computational tools have become indispensable for forecasting interactions, optimizing community composition, and minimizing reliance on empirical trial-and-error. *In silico strategies enable hypothesis generation, simulation of ecological dynamics, and pre-validation of emergent behaviors*, offering a rational foundation for predictive and scalable SynCom engineering.

*Genome-scale metabolic models* (GSMMs) provide comprehensive systems-level maps of organismal metabolism, encapsulating all known enzymatic reactions and transport processes encoded within their genome [30]. When applied to microbial consortia, these models facilitate the simulation of cross-feeding dynamics, resource competition, and metabolic fluxes. *Dynamic flux balance analysis* (dFBA) enhances this approach by incorporating time-dependent metabolite fluctuations and modeling community behavior under varying environmental conditions. It enables the assessment of how metabolic fluxes adjust in response to environmental shifts, thus allowing for the prediction of community behavior over time in non-static conditions [31]. Collectively, GSMMs and dFBA offer a robust framework for modeling microbial interactions and emergent properties in SynComs. Despite their potential, these methodologies remain underutilized in plant microbiomes. However, tools such as COMETS [32], BacArena [33], and KBase [34] are now available to support multi-species modeling efforts.

**Figure 3:**
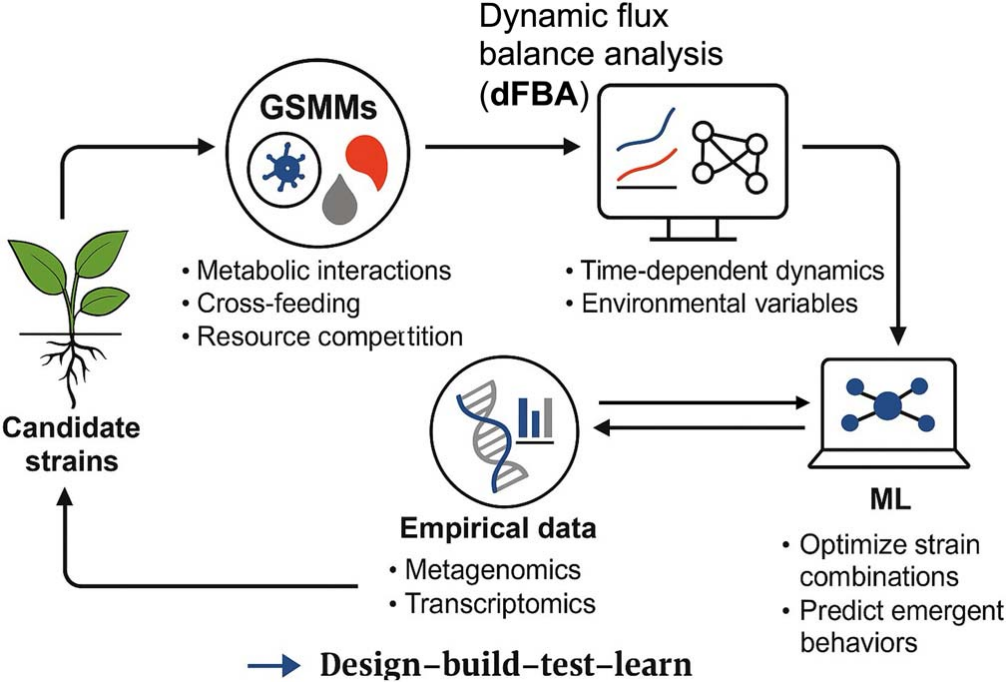
Computational modeling pipeline for rational SynCom design. This schematic illustrates a stepwise framework for in silico engineering of synthetic microbial communities (SynComs) tailored to plant holobionts. GSMMs simulate metabolic interactions, cross-feeding, and resource competition among candidate strains. dFBA integrates time-dependent dynamics and environmental variables. Empirical data (e.g. from metagenomics or transcriptomics) further constrain models, improving prediction accuracy. Machine learning (ML) is used to identify optimal strain combinations and predict emergent behaviors under stress conditions (e.g. drought, salinity). This integrated workflow enables data-informed SynCom design through iterative “design–build–test–learn” cycles, supporting field-ready microbiome engineering.

Recent studies show the power of GSMMs in decoding rhizosphere interdependencies. Multi-genome modeling revealed functional modules in the *Arabidopsis root microbiome* [35], while a framework for trophic inference in crop-native rhizobiomes uncovered keystone interactions and syntrophic loops [36]. These insights remain inaccessible through cultivation-based approaches alone. A comprehensive pipeline has been proposed to integrate these computational and ecological steps into the rational design of SynComs (Fig. 3)*.*

Modeling is most effective when paired with *empirical data* [37]. Coupling GSMMs with metagenomic or transcriptomic datasets refines network constraints and validates predictions [38–40]. *Machine learning* (ML) further enhances this by detecting nonlinear patterns and compositional rules from large datasets, guiding climate-smart SynCom design for drought, salinity, and nutrient stress [41, 42].

While *metabolic models* often emphasize carbon fluxes, ongoing efforts are expanding the model's scope to include signaling, stress responses, and gene regulation. Integrative platforms that merge GSMMs, ML, and ecological theory are advancing “*design-build-test-learn*” workflows tailored to plant holobionts. These systems are not merely predictive—they enable rational, function-driven microbiome engineering.

## Synthetic biology meets microbiome engineering

Synthetic biology revolutionizes microbiome engineering by enabling microbial strains with precisely controlled functions and context-responsive behaviors. Within the plant holobiont, this discipline merges *ecological insights* with *design principles* to modulate microbial activity with robustness and precision.

Modular genetic parts—such as *promoters*, *repressors*, and *riboswitches*—can be assembled into synthetic circuits [43] that mimic *logic gates* [44], *toggle switches* [45], and *feedback loops* [46]. These *programmable circuits* let microbes detect plant signals (e.g. flavonoids, stress hormones) and respond by producing beneficial outputs like antimicrobial peptides or growth regulators [47]. For instance, engineered *Pseudomonas* strains [48] can activate biocontrol genes only in response to plant stress and pathogen cues, enhancing targeted defense while minimizing collateral effects.

*Chassis selection* remains key for field performance [49]. Native strains offer better colonization but are less amenable to genetic editing. In contrast, model strains like *E. coli* [46] or *Bacillus subtilis* [50, 51] are more tractable but may underperform in soil. Domestication of native isolates offers a compromise—combining ecological fitness with engineering compatibility.

Advances in *CRISPR-based transcriptional control*, orthogonal *quorum sensing* systems, and biocontainment strategies (e.g. *kill-switch* [52]) provide layered control over engineered microbes. These tools increase predictability and safety in complex soil environments.

*Synthetic circuits* exemplify the shift toward rational, programmable SynCom design. Box 1 introduces a rhizosphere economics analogy to conceptualize how synthetic biology reshapes plant-microbe interactions through behavioral control and resource negotiation.

Box 1. Rhizosphere Economics – Trade-offs in Microbe–Plant Interactions.

The rhizosphere is a dynamic economic arena where plants and microbes exchange resources and services. Plants act as resource providers, releasing carbon-rich exudates to shape microbial communities. In return, microbes may deliver benefits such as nutrient solubilization, pathogen suppression, or abiotic stress mitigation [53]. However, not all interactions are mutually beneficial, paralleling economic dilemmas like freeloading, market imbalance, and misaligned incentives.

Key trade-offs and economic dynamics include:

*Cooperation vs. Cheating*: Beneficial microbes that invest in public goods (e.g. siderophores, antibiotics) risk exploitation by freeloaders that benefit without contributing. Synthetic biology can help stabilize cooperation through engineered communication systems and private-benefit strategies.

*Short-term vs. Long-term Gain*: Plants may prioritize rapid growth via nutrient acquisition, while neglecting microbial traits that promote long-term resilience. Engineering microbial consortia that balance both timeframes is an ongoing challenge.

*Host Sanctions and Rewards*: Plants can differentially reward helpful microbes (e.g. via targeted exudates or niche access) and sanction non-cooperative ones. Understanding and harnessing these mechanisms is key to designing stable SynComs.

*Opportunity Cost in Exudation*: Root exudation is metabolically expensive. Plants must balance the cost of attracting beneficial microbes with the risk of attracting pathogens or wasting resources in unresponsive soils.

*Market-Like Dynamics*: Microbes compete for host-derived resources, forming microbial “markets.” Supply–demand principles, spatial niches, and feedback loops shape these interactions.

*Economic analogies* help integrate ecological theory with SynCom design. These economic analogies manifest in real-world microbial traits, distinguishing cooperative from opportunistic behaviors. See Table 1 for a side-by-side comparison of these traits across beneficial and pathogenic microbes. Building on this framework, the following section explores how synthetic circuits bring programmable behavior to life within engineered microbial agents.

**Table 1:** Microbial traits shared across beneficial and pathogenic microbes.

**Trait Type**	**Examples in Beneficials**	**Examples in Pathogens**	**Functional Role**
**Secretion systems**	*Type VI secretion* in *Pseudomonas fluorescens* [48]	*Type III secretion* in *Ralstonia solanacearum* [53]	Modulate host immunity, intermicrobial competition
**Effector proteins**	Modulate root architecture (e.g. *Nod factors*) [53]	Suppress PTI via Avr proteins [53]	Alter host physiology or evade immune recognition
**Small molecule signals**	Volatiles, auxins, siderophores [54]	Toxins, coronatine [54]	Influence host signaling, stress tolerance, or cell damage
**Quorum sensing**	Regulates biocontrol gene expression [55, 56]	Controls virulence expression (e.g. *luxR*) [55]	Synchronizes microbial behavior in plant environments
**Motility & chemotaxis**	Root colonization by *Bacillus subtilis* [26]	Pathogenesis by *Xanthomonas* spp. [53]	Navigate toward host-derived signals and niches
**Biofilm formation**	Protective consortia by *Rhizobium* spp. [53, 57]	Persistent infection structures [53]	Enhance survival and facilitate host interaction
**Cell wall-degrading enzymes**	Release nutrients from rhizodeposits [53]	Breach host tissues (e.g. pectate lyases) [53]	Enable resource acquisition or pathogenesis

## Programmable microbial agents and gene circuits

**Figure 4:**
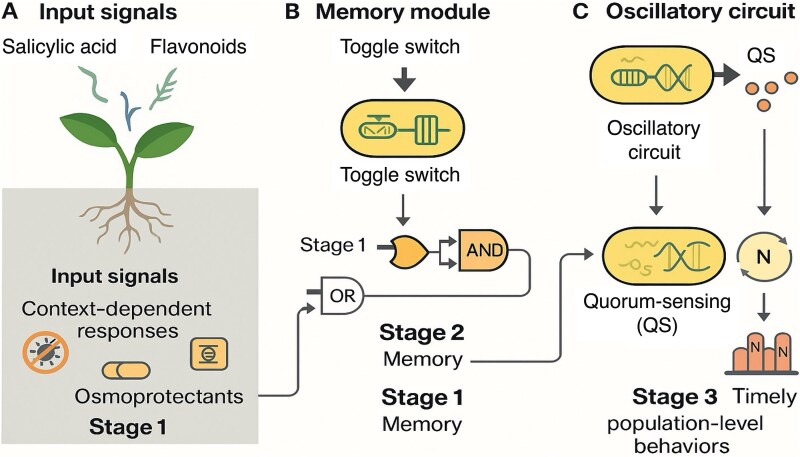
Synthetic gene circuits for signal-responsive behavior in rhizosphere microbes. Engineered microbes integrate environmental and plant-derived cues using modular genetic circuits. (A) Input signals such as salicylic acid and flavonoids are detected in the rhizosphere and drive context-dependent microbial responses, including production of osmoprotectants or antimicrobial compounds (*Stage 1*). (B) Memory modules such as toggle switches process and store transient signals, enabling microbes to maintain persistent gene expression patterns. Logic gates (e.g. AND, OR) allow conditional activation depending on combinations of cues, supporting programmable microbial memory (*Stage 2*). (C) Oscillatory circuits coupled with quorum sensing (QS) enable population-level coordination, regulating behaviors such as biofilm formation, sporulation, or metabolite secretion in sync with environmental or plant rhythms (*Stage 3*). This synthetic logic architecture enables precise and dynamic microbial responses within the plant holobiont.

One of the most groundbreaking advancements in microbiome engineering is the creation of *synthetic gene circuits*, which allow microbes to function like biological computers [58]. These circuits can detect cues from the environment or the host, perform logic operations, and activate specific outputs only when certain conditions are satisfied. Engineered constructs have been developed to sense plant signals, compute logic operations, and trigger rhizosphere responses through modular design elements such as biosensors and logic gates (Fig. 4).

Using *genetic logic gates* (e.g. AND, OR, NOT), microbes can be programmed to respond only to combinations of cues, such as root exudates, stress hormones, or quorum-sensing molecules [55, 56, 59–61]. For instance, a bacterium may secrete antimicrobials only when a pathogen signal and a plant stress hormone are present, minimizing off-target effects on non-pathogenic taxa.

Advanced circuits also include *memory modules* [62, 63], such as *toggle switches* and recombinase-based systems, which allow microbes to “remember” transient signals like drought or salinity. This enables a switch to persistent stress-response states, supporting plant resilience and contributing to holobiont-level adaptation.

*Oscillatory circuits* and *synchronized clocks* offer further precision [46, 64]. These have coordinated nitrogen fixation and biocontrol expression with plant diel rhythms in model systems [65]. Coupling such circuits with *quorum sensing* allows microbial populations to assess density and adjust behaviors—e.g. biofilm formation, or metabolite secretion—only when ecologically viable [57, 66].

This logic-based architecture supports the development of programmable, responsive microbes embedded within plant holobionts. As these circuits become more robust and compatible with native chassis, they will enable *next-generation programmable and self-regulating bioinoculants*.

## Expanding control of microbial behavior

Controlling microbial behavior in complex environments requires genetic *programming*, *multi-layered regulation*. Advances in synthetic biology enable engineered microbes to adapt, coordinate, and self-regulate within the holobiont.

One major innovation is using *orthogonal quorum-sensing systems* [67], which allow engineered strains to communicate without interfering with native microbes. These systems enable division of labor in SynComs—e.g. triggering biofilm formation or nitrogen fixation only when a threshold population density is reached. Crosstalk is minimized through synthetic AHLs—*signaling molecules* used in quorum sensing, a process by which many Gram-negative bacteria communicate based on population density, or non-native peptide signals.

*Kill switches* [68] and *biocontainment circuits* [69] add a safety layer, permitting conditional termination of engineered strains. Cells can be programmed to self-destruct upon loss of plant contact or in specific soil chemistries, preventing persistence or horizontal gene transfer.

Efforts to integrate *environmental sensors* and *feedback loops* are also advancing [70, 71]. Biosensors for soil pH, redox potential, or nutrient levels can regulate gene expression, metabolic output, or motility, enabling context-aware interventions that dynamically support plant health.

Inducible systems have also enhanced external control over microbial activity. Light-inducible switches (optogenetics) [72, 73], nutrient-responsive promoters [74], and temperature-sensitive regulators [75] allow researchers or farmers to spatially and temporally control microbial gene expression, enabling new applications in *precision agriculture*.

Altogether, these tools *shift microbial inoculants from passive agents to active participants*, capable of real-time environmental sensing, adaptive behavior, and engineered interactions. Such dynamic control is essential for creating SynComs that are functional, safe, predictable, and robust under field conditions.

## Chassis microbes and advanced engineering

A key enabler of predictable SynCom function is the availability of microbial chassis that are genetically tractable, ecologically compatible, and stable in the field. While model strains such as *E. coli* [48] and *B. subtilis* [58] have been invaluable in developing synthetic circuits [76], their poor competitiveness in the rhizosphere limits their use as chassis in agricultural systems. Developing *native chassis*—microbial strains naturally associated with plants and adapted to soil ecosystems—is therefore an emerging frontier in holobiont engineering.

The ideal chassis microbe must balance robustness with engineering flexibility. Native strains like *Pseudomonas fluorescens*, *Rhizobium leguminosarum*, and *Bacillus velezensis* have demonstrated plant-beneficial traits and are increasingly explored for their compatibility with genetic manipulation [77]. *Genome reduction* strategies are employed to streamline metabolic burden and remove redundant or mobile elements, increasing predictability and reducing ecological risk.

Advancements in genome editing, particularly CRISPR-Cas systems, have facilitated the rapid modification of candidate chassis. These tools allow for targeted knock-ins, knock-outs, and modular pathway insertion without compromising native fitness. Additionally, synthetic genome refactoring [78]—where genes and regulatory elements are reorganized for greater control—has begun to reshape native microbial genomes to align with design goals.

Another consideration is horizontal gene transfer (HGT) and genetic stability [79]. Engineered traits must remain stable over time and across environmental conditions, avoiding dissemination to non-target organisms. Strategies such as site-specific recombination, toxin-antitoxin systems, and active partitioning systems are being integrated to limit HGT and ensure biosafety [79–81].

The development of standardized microbial chassis parallels trends in synthetic biology, where design rules are being codified, and repositories of well-characterized parts are expanding. In the context of the *plant holobiont*, this means assembling functional modules and ensuring that the chassis themselves are field-hardened and capable of long-term colonization and interaction with host plants. Selecting and engineering microbial chassis requires integrating ecological fitness with design modularity, computational foresight, and robust control systems. Supplementary Table S2 synthesizes these principles and outlines strategies and modeling tools supporting chassis-based SynCom design.

## Frontiers and nobel-scale discoveries

The convergence of plant microbiome research and synthetic biology is pivotal for groundbreaking discoveries with profound implications, extending from sustainable agricultural practices to fundamental biological understanding [82]. Several promising avenues in this field hold the potential to yield breakthroughs worthy of significant scientific recognition [83, 84].

One promising area is the development of *transgenerational microbiome engineering* [85]. Early evidence suggests that microbial communities associated with plants can transmit certain traits across generations via seeds or root-associated inheritance [86]. If validated and harnessed, this mechanism could allow for long-term trait stabilization in crops without continuous reapplication of beneficial microbes, *redefining the concept of “breeding” at the holobiont level*.

Another frontier is the *synthetic reconstitution of complete plant holobionts in gnotobiotic systems* [87], enabling controlled testing of evolutionary, ecological, and functional hypotheses. The modular construction of synthetic ecosystems in the lab, followed by their functional testing under semi-field conditions, is becoming increasingly feasible with mini-rhizotron systems and microcosm-integrated sensors [88]. These efforts uncover emergent properties of microbe–plant–soil interactions that cannot be predicted from individual parts alone.

A third area of transformative promise lies in *cross-kingdom communication engineering*, where synthetic receptors, signal mimics, and communication bridges are constructed to enable programmable responses between plants and microbes [89–91]. Synthetic auxin sensors, RNA-based information transfer, and CRISPR-based logic integration between microbial and plant gene circuits open new dimensions in plant–microbiome dialogue.

Integrating *microbiome dynamics with climate resilience strategies* also represents a compelling frontier. Engineered microbial consortia that adaptively modulate plant hormonal responses under extreme conditions—drought, salinity, or heat—offer a pathway to stabilize yields in marginal lands. Such developments respond directly to global food security challenges and are at the heart of the *Sustainable Development Goals*.

These frontiers demonstrate how engineering the plant holobiont moves beyond an incremental practice into a transformative scientific endeavor. As synthetic biology, microbial ecology, and plant sciences converge, the stage is set for Nobel-scale discoveries—those that fundamentally reshape our relationship with nature and redefine the unit of biological innovation.

## The programmable holobiont

The *programmable holobiont concept* signifies an innovative integration of synthetic biology, microbial ecology, and systems engineering. Rather than passively shaping microbiome composition through traditional breeding or inoculation, this approach *aims to actively rewire interkingdom interactions* through logic-based control systems and modular design principles.

At its core, a programmable holobiont integrates genetically engineered microbial consortia with plant hosts capable of sensing and responding to microbial signals. By incorporating biosensors, feedback loops, and synthetic gene circuits, microbes can be designed to detect plant-derived cues—such as root exudates [92] or stress phytohormones [93]—and trigger specific functional responses, including nitrogen fixation, antifungal compound production, or modulation of host immunity.

A key advancement is the potential for *bidirectional communication*. Engineered plants can express synthetic receptors or transcriptional regulators that decode microbial signals, allowing the plant to fine-tune its response based on microbial behavior. For example, plant roots may increase carbon flow or reduce immune surveillance in response to the presence of beneficial biosensor-equipped microbes, fostering mutualism through dynamic feedback.

This integration of biological computation in plants and microbes has led to the emergence of *“smart” holobionts*—multi-organismal systems that adaptively coordinate metabolism and defense in response to fluctuating environments. This is particularly valuable in agricultural systems exposed to variable abiotic stressors or unpredictable pathogen outbreaks.

**Figure 5:**
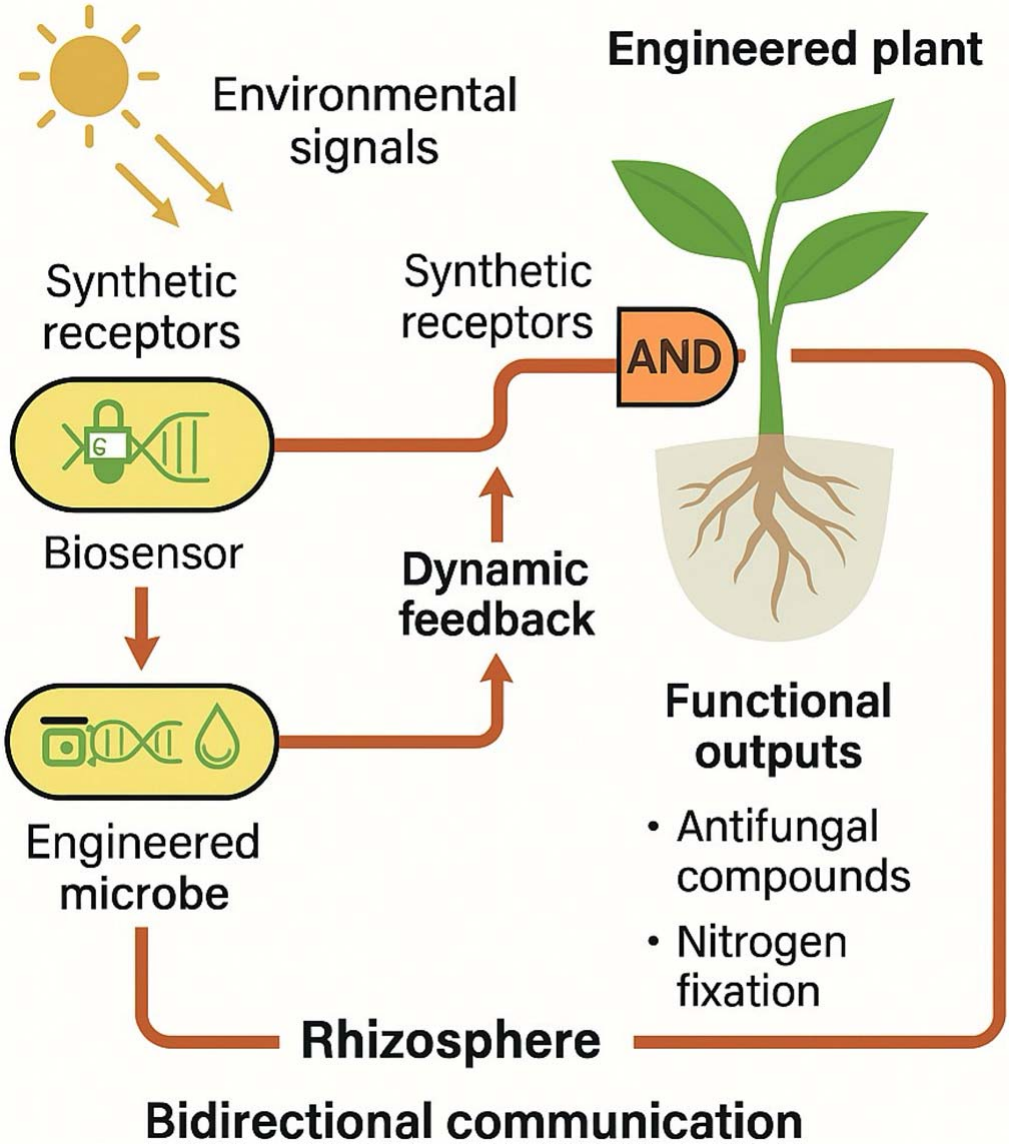
The programmable holobiont: bidirectional logic and adaptive feedback. Conceptual model illustrating engineered plant–microbe interactions governed by synthetic logic and dynamic feedback. Microbial biosensors detect plant-derived signals such as root exudates and stress phytohormones, activating genetic circuits that trigger functions like nitrogen fixation, antifungal compound production, or immune modulation. Engineered plants, in turn, sense microbial signals via synthetic receptors or CRISPR-based regulators, adjusting carbon allocation and immune tone in response. Environmental inputs further shape behavior in both partners. Feedback-regulated root exudation reinforces coordination, establishing a closed-loop, co-adaptive communication system. This programmable holobiont exemplifies multi-organismal integration, where real-time, logic-driven interkingdom dialogue enables system-level resilience and functional innovation.

Integrating sensors, feedback loops, and logical coordination between plant and microbe leads to a *programmable holobiont system* (Fig. 5)*.* Thus, the programmable holobiont embodies a new unit of biological innovation. Moving beyond the engineering of isolated traits sets the foundation for whole-system design, where function emerges from the synergistic behavior of modular, interacting biological agents.

## Rewiring inter-kingdom communication

Plants and microbes naturally exchange molecular signals that coordinate development, immunity, and metabolism. These signals include phytohormones, volatile organic compounds (VOCs), small RNAs, and peptides. Synthetic biology now enables the rewiring and enhancement of these communication channels to increase *bandwidth*, *specificity*, and *control* in plant-microbe interactions.

One promising strategy involves engineering microbial biosensors that selectively detect plant-derived signaling molecules and trigger context-specific physiological responses. For example, synthetic promoters responsive to compounds like salicylic acid [94], jasmonic acid [54], or root exudates such as coumarins [95] could be integrated into native microbial strains [96] such as *Pseudomonas* and *Bacillus*. This strategy would enable the targeted activation of traits such as biofilm formation, sporulation, or antibiotic production, specifically within the rhizosphere environment.

Plants can also be engineered to recognize microbial signals with greater sensitivity and precision. A recent synthetic system enabled plants to detect bacterial “sender” molecules (e.g. *p*-coumaroyl-HSL) via engineered receivers, triggering *context-dependent responses* [82]. Depending on the environmental context, plants use custom receptors or CRISPR-based transcription factors to detect quorum-sensing molecules or synthetic peptides and respond via stomatal closure, auxin modulation, or ROS signaling.

Bidirectional communication modules can also be designed with *logic gate architectures* (e.g. AND, NOT) to ensure responses occur only under specific signal combinations. For instance, a defense pathway might activate only when microbial density is high and the plant is under drought stress, improving ecological safety and reducing off-target effects.

These systems signify a transition from static inoculants to *dynamic synthetic ecosystems*, wherein plants and microbes engage in *real-time molecular dialogue*. This interaction allows the holobiont to coordinate adaptive responses and optimize shared ecological outcomes effectively.

## Microbiome memory and transgenerational inheritance

A growing frontier in plant holobiont research is the idea of *microbiome memory*—that plants and their associated microbes can retain information about past environmental or biotic interactions, influencing future responses. This “*memory”* may be encoded in community composition, microbial gene expression, or host epigenetic and metabolic priming [97].

Abiotic stresses (e.g. drought, salinity) or pathogen exposure can selectively enrich protective microbes in the rhizosphere. These shifts often persist through plant development or even across generations. Progeny of stressed plants frequently harbor altered microbiomes and exhibit enhanced resistance [98], suggesting that *adaptive microbial configurations* can be transmitted via seeds or vegetative propagation [99]. Engineering endophytes for seed colonization is a promising strategy, as seen in grasses and ongoing work in legumes and cereals.

**Figure 6:**
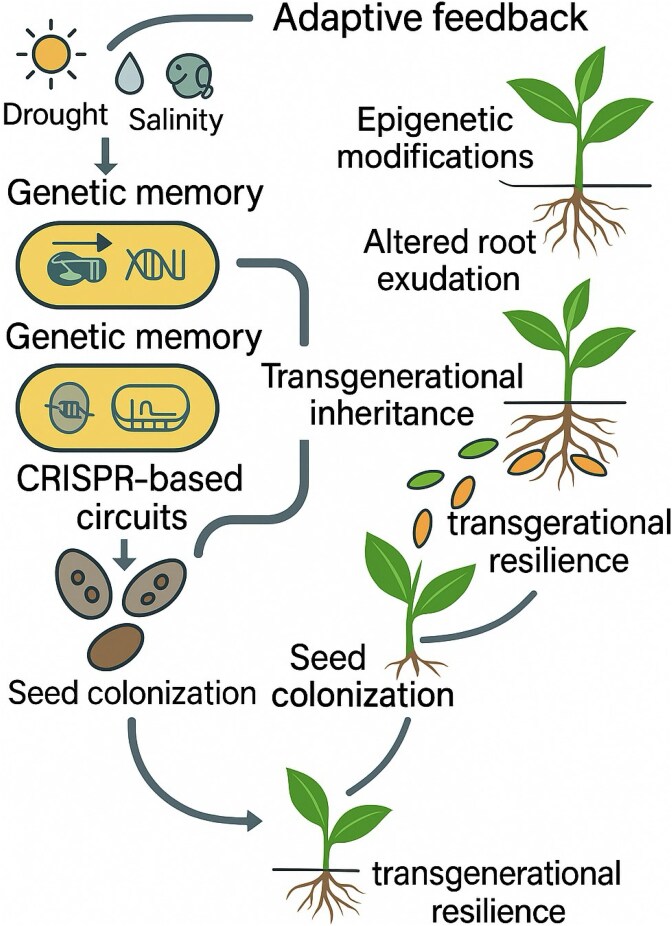
Microbiome memory modules and transgenerational inheritance in engineered holobionts. Schematic diagram illustrating how microbiome memory enhances plant resilience across generations. Engineered microbes integrate environmental stress signals (e.g. drought, salinity, pathogen exposure) through genetic memory modules (toggle switches, CRISPR-based circuits), maintaining persistent altered states even after transient stresses. These microbes colonize seeds, transmitting adaptive microbiomes vertically. Simultaneously, plants encode stress responses through epigenetic modifications and altered root exudation profiles, shaping microbiome composition across generations. This dual-layered “holobiont memory” integrates microbial and plant mechanisms, fostering ecological stability, resilience, and adaptive capacity.

*Microbiome memory* can also be synthetically programmed. Toggle switches and CRISPR-based memory modules enable engineered microbes to “record” specific environmental cues—such as stress signals or exudates—and maintain a persistent altered state even after the trigger fades. These systems can prime future plant generations, reducing the need for repeated microbial inoculation by enhancing transgenerational resilience and *holobiont-level information storage* (Fig. 6).

Parallel, *plant-side mechanisms*—including epigenetic inheritance and stable changes in root exudation profiles—also contribute to memory [100]. Plants may be engineered to recruit specific microbial allies or consistently reinforce cooperation across generations. Integrating plant and microbial memory creates a new concept of *synthetic holobiont inheritance*, where resilience and ecological experience become embedded in biological structure. This could enable crops to adapt to current environments and evolve anticipatory responses to future stressors.

## Challenges in translating lab successes to field conditions

Despite significant strides in synthetic biology and microbiome engineering, *translating lab-based innovations into field-ready applications* remains a key challenge. Synthetic communities (SynComs) and engineered microbes often fail to perform under the heterogeneous, unpredictable conditions of agricultural soils.

One major obstacle is the *complexity and competitiveness of natural microbiomes*. Real-world soils host thousands of taxa engaged in dynamic interactions [101, 102], rarely reflected in simplified SynComs of 10–100 strains. These engineered consortia often lack *ecological resilie*nce and are quickly outcompeted or lose functionality. Environmental variability—soil heterogeneity, climate fluctuations, and root architecture—further complicates microbial establishment and persistence.

The effectiveness of microbial agents is highly context-dependent, with outcomes shaped by crop genotype, soil conditions, and agricultural practices. A strain that performs well in one environment may underperform or disrupt ecological balance in another. These limitations have prompted efforts toward *localized microbiome design*, favoring native strains with adaptations to specific field contexts. For example, a tomato rhizosphere isolate—*Pseudomonas putida* KT2440—outperformed non-native strains in delivering drought tolerance traits [103]. Still, scalability and reproducibility remain significant barriers to widespread application.

Regulatory and biosafety concerns further restrict field deployment. Engineered microbes face strict oversight in many countries. Though trials of engineered *P. fluorescens* showed no harmful effects, fears of horizontal gene transfer and ecosystem disruption persist [104]. Biosafety tools, including kill switches, nutrient-dependent circuits, and adaptations of GURTs (“terminator” technologies) [105], are being explored to enhance containment. Nevertheless, regulatory frameworks often focus on single organisms rather than microbial communities [106]. New policies—aligned with the *Nagoya Protocol* and SynCom-specific guidelines—are needed for holistic risk assessment and equitable bioprospecting.

Bridging the lab-to-field gap will require *multi-pronged strategies*: pre-screening SynComs in semi-natural conditions, incorporating persistence traits (e.g. sporulation, biofilms), and using carriers like alginate beads [106], lyophilized matrices, or seed coatings for stable delivery [107]. These techniques improve microbial survival and enable co-establishment with crops.

Ultimately, the successful deployment of synthetic holobionts will depend on technological, ecological, and regulatory integration and public trust. Cross-sector collaboration among scientists, agronomists, and policymakers will be critical to realizing the potential of microbiome engineering in climate-smart agriculture.

## Future directions

Engineering the plant holobiont to enhance agricultural resilience necessitates *a comprehensive roadmap* that amalgamates biological understanding, technological advancement, and ecological pragmatism. Future developments will hinge on implementing strategies across three interrelated dimensions: plant-centered, microbe-centered, and holobiont-centered interventions.

### Plant-centered strategies

Efforts to optimize root exudation profiles, stress signaling pathways, and immune priming are critical for shaping microbiome assembly. *Precision breeding* and *genome editing* (e.g. CRISPR, base editing) can be used to design plants with enhanced capacity to recruit and maintain beneficial microbes, even under adverse conditions. Additionally, engineering exudate-mediated selection pressures may help guide microbiome trajectories in a host-specific manner.

### Microbe-centered strategies

The future of SynComs lies beyond taxonomic selection—it will be driven by functional design. Modular microbial traits (e.g. nitrogen fixation, phytohormone production, pathogen suppression) can be reprogrammed through synthetic biology. Mobile genetic elements and CRISPR-based systems offer tools to insert or regulate key functions. Further, developing computational models that predict performance under variable field conditions will enable a more robust SynCom assembly.

### Holobiont-centered strategies

Holistic integration of plant and microbial traits is required to construct dynamic, adaptable holobionts. *Holobiont breeding* aims to select plants not only for yield or disease resistance, but also for their ability to support beneficial microbiomes. This includes screening germplasm for varieties that foster high-performing microbial consortia, even in low-input systems. For example, maize lines that enhance phosphorus-solubilizing bacterial populations can be prioritized, and QTL mapping reveals genetic loci tied to root exudates that shape these communities.

Future holobiont designs will likely include engineered feedback loops, where plant signals modulate microbial functions and vice versa. Co-evolutionary algorithms and agent-based simulations may support the design of responsive learning ecosystems—biological systems that improve over time through memory and feedback.

### Beyond the bench

Field-deployable biosensors, AI-guided microbiome analytics, and responsive agricultural practices will form the support systems of next-generation applications. Equally important are the social, economic, and ethical frameworks that govern deployment. Participatory design involving farmers, regulators, and local communities is essential to ensure scalability, equity, and public acceptance.

## Path forward: integrating innovation with ecological realism

The future of plant holobiont engineering lies in navigating the *delicate balance between innovation and ecological realism*. While synthetic biology and computational modeling provide unprecedented control over microbial traits and interactions, their field success will ultimately depend on context-aware design, regulatory foresight, and systems-level validation.

A critical path forward involves adopting adaptive design principles and viewing synthetic communities (SynComs) and engineered microbes not as static instruments but as dynamic constituents of living ecosystems. This approach entails integrating feedback circuits, redundancy, and resilience within design frameworks, such as *strategies for ecological insurance*. Drawing from systems ecology, future interventions should prioritize functional robustness over taxonomic precision, acknowledging the inherent plasticity of microbial roles. Moreover, effective implementation will require *interdisciplinary collaboration* among synthetic biologists, microbial ecologists, plant breeders, agronomists, and policy experts. A modular pipeline—from lab screening to greenhouse testing to field trials—must become the norm, with each step accompanied by ecological metrics and risk evaluation. Synthetic biology cannot remain siloed in the lab; its outputs must be validated in the messy reality of the soil.

Involving farmers can uncover Indigenous knowledge that sparks innovative synthetic community designs. It is also essential to distinguish “*bioengineering of microbiomes*” from traditional *Genetically Modified Organisms*. Engaging local communities in biodiversity-rich areas is vital, as they often hold valuable microbial resources.

Crucially, *governance frameworks* must evolve alongside the science. This involves harmonizing biosafety protocols, standardizing approval processes for engineered strains, and facilitating equitable access, particularly within the Global South. Integrating ethical foresight and transparent communication into research frameworks will enhance public trust and contribute to long-term sustainability.

In sum, the path forward is neither purely technological nor purely ecological—it is both. Engineering plant holobionts for the Anthropocene will demand humility, adaptability, and a shift from domination to co-creation with nature. Only by *integrating innovation with ecological realism* can we realize the full potential of synthetic biology for resilient, equitable agriculture.

## Conclusion

Plants exist in a microbial continuum where friends and foes share molecular signatures and where outcomes depend on the context of host perception and resource availability. As our understanding of the plant holobiont deepens, we are now entering an era in which we can *move beyond descriptive microbiome research toward active design and engineering*.

This review has highlighted how tools from synthetic biology, computational modeling, and systems ecology are converging to shape a new discipline—one that seeks to engineer resilient, responsive, and ecologically sound plant-microbiome systems. From SynCom design and metabolic modeling to programmable gene circuits and chassis optimization, we are learning to harness microbial functionality for yield gains, adaptive resilience, ecosystem sustainability, and climate-smart agriculture.

*Transitioning from lab innovation to field implementation* remains a significant hurdle. Future work must balance innovation with realism, control with adaptability, and technological precision with ecological understanding. The holobiont is not merely a system to be engineered—it is a partner in co-creation, embedded in soil, season, and socio-political context.

By integrating lessons from pathogens and symbionts and adopting flexible, inclusive design frameworks, we can begin to translate this vision into action. The programmable holobiont is not a distant ideal—an emerging reality key to transforming agriculture in the Anthropocene.

## Supplementary Material

Supplementary_Table_1_revised_wraf158

Supplementary_Table_2_revised_wraf158

## Data Availability

Data sharing does not apply to this article as no datasets were generated or analysed during the current study.
