# Supplementary material for: Engineering plant holobionts for climate-resilient agriculture
Source: ISME J. 2025 Aug 1;19(1):wraf158. doi: 10.1093/ismejo/wraf158 (PMC12381762; doi:10.1093/ismejo/wraf158)
Supplement: Supplementary_Table_1_revised_wraf158 [file supplementary_table_1_revised_wraf158.docx]

### **Supplementary Table 1** **| Glossary of Key Terms in SynCom Engineering**

A curated glossary defining core concepts in synthetic community (SynCom) design, engineering, and deployment. Terms include: chassis strain, trait modularity, functional redundancy, CRISPR interference, toggle switch, dFBA, and more. This resource provides accessible reference points for readers less familiar with synthetic biology terminology.

| **Term** | **Definition** |
| --- | --- |
| **SynCom (Synthetic Community)** | A deliberately assembled microbial consortium designed for controlled studies or functional purposes. SynComs may be used in *host-associated systems* (e.g., plant microbiome engineering) or in *host-free settings* (e.g., bioreactors for compound production). |
| **Chassis Microbe** | A genetically tractable microorganism used as a host for engineered genetic elements, often selected for colonization ability and safety (e.g., *Pseudomonas fluorescens*, *Bacillus subtilis*). |
| **Keystone Taxon** | A microbial species that exerts a disproportionately large effect on community structure or function relative to its abundance. |
| **Priority Effects** | Ecological phenomena where the order of species arrival influences subsequent community assembly and dynamics. |
| **Modular SynCom** | A SynCom structured around functional modules (e.g., ISR induction, nutrient solubilization), allowing trait-specific customization. |
| **EcoFAB** | An engineered, standardized microcosm system for controlled study of plant–microbe interactions under reproducible conditions. |
| **Microbiome Plasticity** | The ability of a microbial community to adaptively alter its structure in response to environmental or host-associated changes. |
| **Precision Microbiome Editing** | Targeted manipulation of microbiome composition or function using tools like CRISPR, antimicrobials, or phage-based interventions. |
| **Microbial Biosensor** | An engineered microbe capable of detecting environmental signals and reporting them via fluorescence, color change, or other measurable output. |
| **Quorum Sensing (QS)** | A cell-density–dependent communication system in bacteria that regulates gene expression and behavior coordination within microbial communities. |
