# Supplementary material for: Engineering plant holobionts for climate-resilient agriculture
Source: ISME J. 2025 Aug 1;19(1):wraf158. doi: 10.1093/ismejo/wraf158 (PMC12381762; doi:10.1093/ismejo/wraf158)
Supplement: Supplementary_Table_2_revised_wraf158 [file supplementary_table_2_revised_wraf158.docx]

### **Supplementary Table 2 | Tools and Design Principles for Microbiome Engineering**

This table summarizes key design strategies and computational tools used in the rational engineering of microbiomes. It includes principles such as top-down versus bottom-up approaches, trait modularity, niche complementarity, resilience mechanisms, predictive modeling, and representative tools such as dFBA and agent-based models.

| **Concept / Strategy** | **Description** | **Representative Reference(s)** |
| --- | --- | --- |
| **Trait modularity** | Modular traits (e.g., nitrogen fixation, IAA production) can be inserted into SynCom members or plant hosts in plug-and-play fashion. | 45; 83 |
| **Succession** | Rational SynComs consider temporal shifts in microbial composition across plant developmental stages. | 86; 87 |
| **CRISPR interference (CRISPRi)** | Used for precision gene silencing in non-model or synthetic chassis strains. | 54 |
| **Dynamic flux balance analysis (dFBA)** | Simulates metabolite exchanges and growth in complex microbiomes under spatiotemporal constraints. | 34; 38 |
| **Mobile CRISPR arrays** | Programmable mobile systems for targeted modulation of microbial communities or in situ genome editing. | 81 |
| **Microbial guild** | Functional consortia designed around specific nutrient cycles or plant phenotypes. | 41 |
| **Niche complementarity** | Engineered SynComs exploit non-overlapping niches (root tip vs elongation zone, rhizoplane vs endosphere). | 85 |
| **Functional redundancy** | Increases resilience by incorporating microbes with overlapping functions (e.g., multiple phosphate solubilizers). | 42; 101 |
| **Chassis strain** | Standardized microbial hosts (e.g., *Bacillus subtilis*, *Pseudomonas fluorescens*) for circuit integration. | 26; 51 |
| **Minimal microbiome** | SynComs reduced to the lowest number of strains that maintain host benefits. | 84 |
| **In situ modulation** | Strategies that activate microbial traits (e.g., ISR) only in presence of host cues or stress. | 49 |
| **Top-down vs. bottom-up design** | Top-down = simplified SynComs from natural microbiomes; bottom-up = de novo combinations of known strains. | 99 |
